# Oestradiol and breast cancer prevention: a 40 year history and contemporary perspective

**DOI:** 10.1038/s41416-025-03115-0

**Published:** 2025-09-19

**Authors:** Jack Cuzick, Mitch Dowsett

**Affiliations:** 1 John Snow Emeritus Professor of Epidemiology, Wolfson Institute of Population Health, Queen Mary University of London, London, UK; 2 Professor Emeritus, Royal Marsden Hospital, London, UK

**Keywords:** Cancer, Predictive markers

## Abstract

Nearly 40 years ago one of us published a conceptual article arguing the case for studies of breast cancer prevention with tamoxifen. Recent observations on the interaction of plasma oestrogen levels with the preventive effect of the aromatase inhibitor anastrozole make it timely to consider the development of oestrogen-targeted prevention of breast cancer and the evidence upon which that development was made. In this article we review the understanding of the aetiology of breast cancer in the mid-1980s; its subsequent development, including the findings from the adjuvant treatment of early breast cancer with tamoxifen and from the trials that assessed tamoxifen as a preventive agent in women at increased risk. We then focus on results from the comparative trials of aromatase inhibitors (AIs) versus tamoxifen and the extension of the use of AIs in trials for prevention. We describe the relationship between plasma levels of oestradiol and its major protein binder, sex hormone binding globulin, on the development of oestrogen receptor positive breast cancer and how these impact on the preventive effect of the AI, anastrozole, in postmenopausal women. Lastly, we speculate on the potential role of routine measurement of oestradiol in managing risk and achieving prevention of breast cancer.

## Endocrine aetiology of breast cancer in 1986

By the mid-1980s, several epidemiologic observations provided circumstantial evidence that high and or prolonged exposure of the breast to oestrogenic stimulation was likely to increase the risk of breast cancer. It was known that early age of menarche and late age of menopause, and thus lifetime exposure to ovarian hormones, were both related to an increased risk of developing breast cancer [1]. It was also known that nulliparous women and those who had a late age at first childbirth had a higher risk of breast cancer than parous women, although the way this might relate specifically to oestrogens was unclear [2]. This association with breast cancer had been based on much earlier observations by the Italian physician and father of occupational medicine, Bernardino Ramazzini (1633–1714) cited by Olson [3], and those of Rigoni-Stern (1810–1855) [4], who noted the high rate of breast cancer in nuns (1842, translated by de Stavola, 1987). The finding that ovarian ablation for non-breast cancer reasons was associated with a 75% reduction in subsequent breast cancer risk provided the most persuasive evidence for a direct relationship with ovarian hormones and risk [5].

**Fig. 1:**
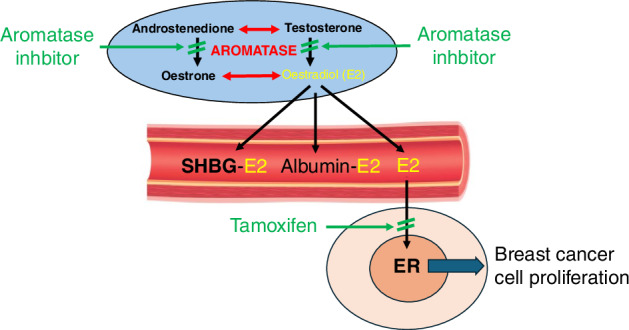
In postmenopausal women oestrogen synthesis from androgens occurs largely in the stromal cells of subcutaneous fat (blue colour). Oestradiol passes into the circulation where the large majority is bound to albumin (weakly) and to SHBG (strongly). Only the protein-free fraction can pass across the cell membrane and therefore be able to bind to the oestrogen receptor (ER) in target cells, such as those of breast cancers, where it drives proliferation. Aromatase inhibitors lead to nearly complete ablation of oestrogen synthesis at source while tamoxifen blocks the binding of oestradiol to ER.

Multiple studies comparing the level of plasma oestrogens in breast cancer patients versus controls without breast cancer provided a variable set of results, probably due at least in part to confounding factors that impact on oestrogen levels after a breast cancer diagnosis. However, the results from a pioneering prospective collection of plasma from women in Guernsey showed that women with higher proportions of the non-protein-bound fraction of oestradiol, ie the biologically active fraction, had a greater risk of developing breast cancer [6]. The comprehensive results of Key et al. [7] strongly confirmed these important results. Plasma oestradiol binds weakly to albumin but much more strongly to Sex Hormone Binding Globulin (SHBG), and the level of SHBG is a strong determinant of the proportion of oestradiol unbound to protein (Fig. 1).

## Endocrine treatment and the Initiation of breast cancer prevention studies

Until the 1970s endocrine treatment of breast cancer was based mainly on surgical removal or ablation of the ovaries in premenopausal women, and adrenalectomy or hypophysectomy in postmenopausal women or those previously subjected to oophorectomy. High doses of oestrogens or androgens also led to some clinical responses, but with major unpleasant side-effects. In each of these studies this was restricted to patients with advanced disease. Modern day medical endocrine treatment began with tamoxifen.

Tamoxifen was originally designed as a contraceptive pill in 1962, where it was a failure [8]. However, Arthur Walpole pressed on to develop it for breast cancer treatment, initially for advanced disease [9]. In the late 1970s, several clinical trials of post-surgical tamoxifen versus no post-surgical treatment in patients with primary breast cancer were begun. In one of these, Cuzick and Baum [10] made the seminal observation that as well as reducing breast cancer recurrence, 2 years of post-surgical tamoxifen significantly reduced the number of new cancers in the contralateral breast from 10 to 3 (*p* = 0.05), providing the first indication that it might be a useful agent for preventing new primary cancers.

These findings, the relatively good tolerability of tamoxifen noted in the adjuvant trials and the endocrine aetiology of breast cancer, as outlined above, led to the proposal for tamoxifen to be evaluated as a breast cancer preventive in women at high risk (nulliparity or late first childbirth; SHBG levels below the population median; high breast density; family history of breast cancer; previous fibrocystic disease) [11]. This proposal led to a meeting of experts later that year in London to discuss conducting a prevention trial in high-risk women without breast cancer. While there was a strong and increasing interest in the use of endocrine agents for adjuvant treatment, substantial concern was raised at the meeting about giving a drug used to treat breast cancer to women without cancer.

Over the next few years, Trevor Powles led a single centre trial of tamoxifen vs placebo, initially as a small feasibility study that addressed some of the concerns of tamoxifen use regarding lipids and bone loss [12]. This “Tamoplac” study eventually developed into a full scale trial of 2471 high-risk women [13], and three other trials began in the early 1990’s: the International Breast Intervention Study 1 (IBIS-I) [14], the National Surgical Adjuvant Breast and Bowel Project (NSABP) P-1 study [15, 16] and an Italian study [17]. This work was against a background of continued dispute about tamoxifen’s use as a preventative agent, much of if based on opinion and not data. A report that rats given tamoxifen developed liver cancer [18] gained special prominence in the debate, despite the fact that by then a very large number women had by then been given tamoxifen in the adjuvant setting, and there were no reports of an increase in liver cancer or other cancers, except for the well-known increase in endometrial cancer [19].

Over subsequent years tamoxifen has been extensively evaluated and used as a hormonal adjuvant treatment for oestrogen receptor positive breast cancer. An overview of the now large number of trials showed that 5 years’ treatment reduced the risk of breast cancer recurrence by about 40% and the risk of dying from breast cancer by about 30% [20]. The original observation of a reduction in contralateral cancers after adjuvant use of tamoxifen was confirmed with a 38% reduction being reported in the overview. These findings have been remarkably accurate in predicting the impact of tamoxifen in the preventive setting and have supported more studies in adjuvant breast cancer to increase understanding of the potential of this new treatment in the prevention setting.

## Breast cancer prevention trials with tamoxifen

In 1992, NSABP initiated the P-1 trial [15, 16]. A total of 13,388 women at high risk were randomly assigned to receive tamoxifen or placebo for 5 years. After 7 years of follow-up, the reduction in new invasive breast cancer in the tamoxifen arm was 41.6% (Hazard Ratio [HR] = 0.57 (0.46–0.70), *P* < 0.001) based on 145 cases in the tamoxifen arm and 250 in the placebo arm. It appears unlikely that any further follow up will be possible to look at long-term prevention.

**Fig. 2:**
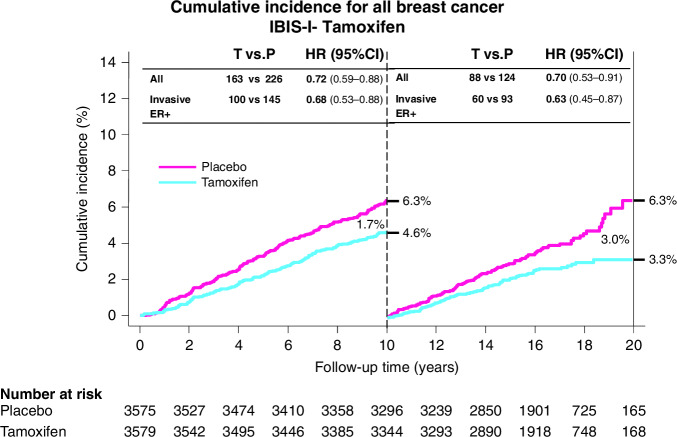
Cumulative risk of breast cancer in the IBIS-I trial comparing tamoxifen with placebo. For the full follow up period the incidence of all breast cancer was 12.6% for placebo and 7.9% for tamoxifen (*p* < 0.0001). For the first 10 years it was 6.3% for placebo and 4.6% for tamoxifen (HR = 0.72, *p* = 0.01) and the ratio was similar subsequently (HR = 0.69, *p* = 0.009).

Also in 1992, International Breast Cancer Intervention Study-1 (IBIS-I) began, a randomised trial of the impact of 5 years of tamoxifen vs placebo on the development of breast cancer in high risk women [14]. High risk was defined as being at least twice the risk of an average risk woman of the same age, based on the Tyrer-Cuzick (IBIS) risk model [21, 22]. A total of 7154 eligible women were recruited from genetics clinics and breast care centres in eight European countries (3579 to tamoxifen and 3575 to placebo). After a median follow up of 16.0 years, 601 breast cancers had been reported: 251 (7.0%) in the tamoxifen arm vs 350 (9.8%) in the placebo arm, equating to a 29% reduction in the occurrence of cancer (HR = 0.71 (0.60–0.83), *P* < 0.0001, Fig. 2) [23]. The benefit in the tamoxifen group was similar in the first 10 years of follow-up (HR = 0.72 (0.59–0.88), *p* = 0.001) to that in subsequent years (HR = 0.69 (0.53–0.91), *p* = 0.009), suggesting a potential life-time benefit from five years of treatment. The greatest reduction in risk was seen in invasive oestrogen receptor positive (ER+) breast cancer (HR = 0.66 (0.54–0.81), *p* < 0.0001) and ductal carcinoma in situ (0.65, (0.43–1.00], *p* = 0.05), but no effect was observed for invasive ER-negative (ER-) breast cancer (HR 1.05. (0.71–1.57), *p* = 0.8).

In an overview of the 4 tamoxifen prevention trials in 2003, Cuzick et al. [24] reported on the major outcomes in 14,192 high-risk women randomised to tamoxifen versus 14,214 randomised to placebo. At that time, including in situ lesions, there were 289 cancers in the tamoxifen arm vs 465 in the placebo arm (38% reduction, *P* < 0.0001). For ER+ invasive cancers there were 135 cases in the tamoxifen arm vs 267 cases in the placebo arm (48% reduction, *P* < 0.0001). There was a small statistically non-significant increase in the incidence of ER- tumours in the tamoxifen arm: 88 vs 72 cases. Venous thromboembolic events were increased in the tamoxifen-treated population (118 vs 62 cases; HR = 1·9 (1·4–2·6), *P* < 0.0001). The rate of endometrial cancer was increased with tamoxifen (53 vs 22 cases; HR = 2·4, (1·5–4·0). *P* = 0.0004), an effect largely confined to those over 50 years of age. In contra*st* to these deleterious effects, a decrease in osteoporotic fractures was observed in the tamoxifen arms.

More recently, studies by two groups have examined whether doses of tamoxifen below the usual 20 mg/d could maintain the drug’s preventive effect but have fewer side-effects. Decensi and colleagues [25] showed that tamoxifen doses as low as 1 mg/day suppressed the proliferative marker Ki67 as much as 20 mg/day did in a presurgical study of ER-positive breast cancer. The same team proceeded to show at 5 mg/day given for 3 years in 500 women aged under 75 years with intraepithelial neoplasia (20% with atypical ductal hyperplasia, 11% LCIS, and 69% DCIS), tamoxifen reduced the development of invasive breast cancer or recurrent ductal carcinoma in situ (DCIS) by 52% compared with placebo (41 vs 66 cases, P = 0.03) after a median follow up of 9.7 years, without additional adverse events [26, 27].

Per Hall and colleagues have looked at the effect of low dose tamoxifen on breast density, a known risk factor for breast cancer, in the Karolinska Mammography project for risk prediction of breast cancer - Intervention Study with Tamoxifen (KARISMA trial) [28]. They studied 1439 women (566 premenopausal, 873 postmenopausal), and offered them tamoxifen at daily doses of 0, 1, 2.5, 5, 10 or 20 mg in a blinded manner for 6 months. They found little reduction in breast density at 0 mg or 1 mg, but reductions similar to the standard 20 mg dose were seen for higher doses in premenopausal women. Limited differences were seen in postmenopausal women. Severe side effects were much less common with 10 mg or less than 20 mg/day.

Overall, tamoxifen appears to be a good choice of drug for premenopausal women. Lower doses seem to be as effective as 20 mg/d and have fewer side effects, but the optimal dose has not yet been determined. Efficacy and tolerability data from trials of aromatase inhibitors indicate that they are a better choice for postmenopausal women.

## Aromatase Inhibitors

Oestrogens are synthesised from precursor androgens by the enzyme aromatase: oestradiol from testosterone and oestrone from androstenedione (Fig. 1). In common with tamoxifen, aromatase inhibitors were first considered for management of reproduction [29]. Through the early 1970s Angela and Harry Brodie compared the effectiveness of large numbers of aromatase inhibitors and selected 4-hydroxyandrostenedione, an irreversible inhibitor as the most potent [30]. In the early 1980s, this became the first specific aromatase inhibitor to be investigated for its use in breast cancer [31]. In the meantime, aminoglutethimide, a relatively non-specific cytochrome P450 inhibitor, was being used in metastatic breast cancer patients because it was thought to act as a “medical adrenalectomy” [32]. However, it was then found that the drug’s effectiveness was independent of its effects on adrenal steroidogenesis and that it was in fact effective in breast cancer due to its inhibition of aromatase [33]. During the 1980s and 90s many aromatase inhibitors were developed for potential use in treating breast cancer, and three eventually emerged for widespread use in both advanced and early ER-positive breast cancer (Arimidex) [34–36], exemestane (Aromasin) [37, 38], and letrozole (Femara) [39], but only anastrozole and exemestane have been used for cancer prevention.

These inhibitors do not suppress oestrogen levels in premenopausal women because enhanced feedback increases the already high levels of the enzyme that exist in the ovary. In postmenopausal women aromatase persists at low levels in peripheral tissues, particularly subcutaneaous fat, and these tissues are responsible for the oestrogen production that continues to drive ER-positive breast cancer after the menopause (Fig. 1). Anastrozole, letrozole and exemestane all lead to near complete inhibition of peripheral aromatase activity in postmenopausal women [40–42]. Their use is sometimes extended to younger women with breast cancer by combination with a GnRH agonist or after chemotherapy-induced amenorrhoea.

## Aromatase inhibitors in early breast cancer

**Fig. 3:**
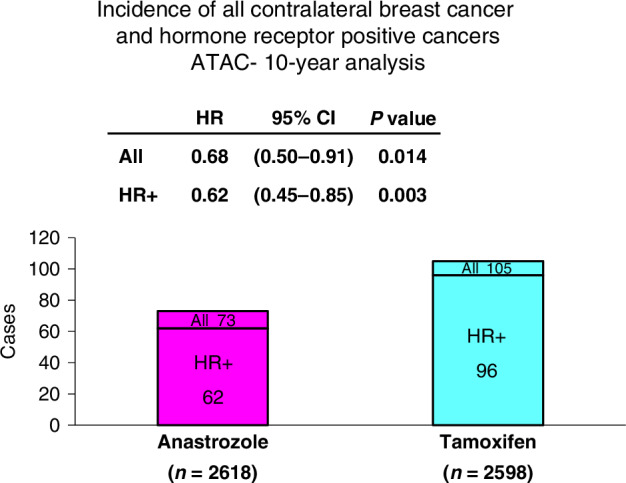
In the ATAC trial anastrozole was compared to tamoxifen in of 9366 postmenopausal women with localised ER-positive cancer. After a 33-month follow up the combination was found to be no better than tamoxifen alone and this arm was dropped leaving a 2 arm trial in 6241 women. After 10 years median follow-up the anastrozole group fared better than those receiving tamoxifen for disease-free survival, time to recurrence and time to distant recurrence. Importantly for prevention, there were fewer contralateral breast cancers with anastrozole, both overall (73 vs 105; HR = 0.68, (0.50–0.91) *p* = 0.014), and for hormone receptor positive cancers (62 vs 96; HR = 0.62, (0.45–0.85), *p* = 0.003).

The ATAC trial (Arimidex, Tamoxifen Alone, or in Combination) was the first major clinical trial of an aromatase inhibitor in early breast cancer. It began in 1996 and compared the effects of anastrozole (1 mg/day) with those of tamoxifen (20 mg/day), alone and those of tamoxifen plus anastrozole [34] for 5 years in a randomized trial of 9366 postmenopausal women with localised ER-positive cancer. After 33.3 months median follow-up the results with the combination were not significantly different from those with tamoxifen alone and the combination arm was discontinued, leaving a two-arm trial of 6241 women. After 10 years median follow-up there were significant improvements in the anastrozole group compared with tamoxifen for disease-free survival (HR = 0·91, (0·83–0·99); *p* = 0·04), time to recurrence (HR = 0·84, (0·75–0·93); *p* = 0·001), and time to distant recurrence (HR = 0·87, (0·77–0·99); *p* = 0·03) [36]. There was weak evidence of fewer breast cancer deaths after recurrence with anastrozole compared with tamoxifen treatment in the hormone-receptor-positive subgroup (HR 0·87, 0·74–1·02; *p* = 0·09), but there was little difference in overall mortality (HR = 0·95, (0·84–1·06); *p* = 0·4). Fractures were more frequent during active treatment in patients receiving anastrozole than those receiving tamoxifen (451 vs 351; HR = 1·33, 95% CI 1·15–1·55; *p* < 0·0001), but were similar in the post-treatment follow-up period (110 vs 112; HR = 0·98, (0·74–1·30), *p* = 0·9). Importantly, especially for prevention, there were fewer contralateral breast cancers with anastrozole than with tamoxifen (73 vs 105; HR = 0.68, (0.50–0.91) *P* = 0.014) (Fig. 3, and treatment-related serious adverse events were less common in the anastrozole group than for tamoxifen (223 anastrozole vs 369 tamoxifen; HR = 0·57, (0·48–0·69), *p* < 0·0001).

Meta-analysis has confirmed the disease-related treatment benefits of aromatase inhibitors over tamoxifen when given for 5 years and followed for 10 years, including a 15% decrease in death from ER-positive breast cancer (HR = 0.85; (0.75–0.96), 2p = 0.009) and a 38% decrease in the risk of contralateral breast cancer (HR = 0.62; 0.48–0.80 2p = 0.0003) [43].

## Aromatase inhibitors in prevention

Given the positive data on anastrozole versus tamoxifen for disease outcome and serious side effects in early breast cancer, clinical trials of aromatase inhibitors given alone as preventive agents have been undertaken.

**Fig. 4:**
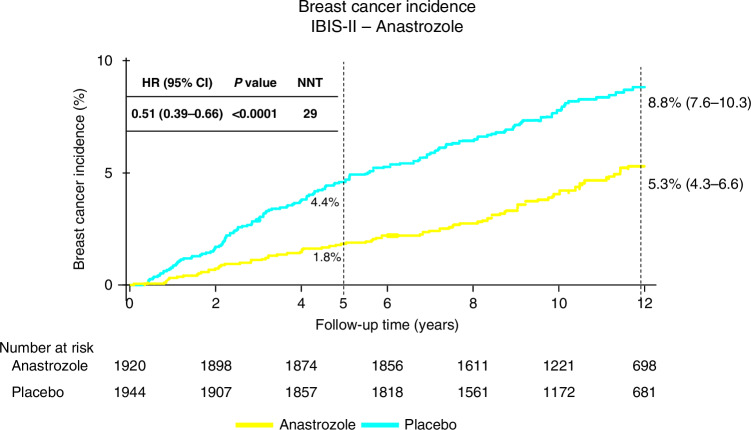
IBIS-II compared anastrozole to placebo in 3864 high risk postmenopausal women. After a median follow-up of 131 months, a 49% reduction in breast cancer was observed in the anastrozole arm (85 vs 165 cases, HR = 0·51, (0·39-0·66), p < 0·0001. The reduction was larger in the first 5 years of follow up (35 vs 89 cancers, HR = 0·39, 0·27–0·58, *p* < 0·0001), but remained significant in the post 5-year period (50 vs 76 new cases, HR = 0·64, 0·45–0·91, *p* = 0·014), and was not significantly different from the first 5 years (*p* = 0·087).

The IBIS-II trial assessed the efficacy and safety of anastrozole for prevention of new breast cancer in postmenopausal women who were at high risk of the disease [44, 45]. In this trial 3864 postmenopausal women at increased risk of breast cancer were recruited between Feb 2003 and Jan 2012 and randomly allocated to either anastrozole (1 mg per day,1920 women) or matching placebo (1944 women) for 5 years. After treatment completion, women were followed annually to collect data on breast cancer incidence, deaths, other cancers, and major adverse events (including cardiovascular disease and fractures). The primary outcome was all breast cancer, including in situ lesions. After a median follow-up of 131 months, a 49% reduction was observed in the anastrozole arm (85 vs 165 cases, HR = 0·51, (0·39-0·66), p < 0·0001 (Fig. 4) [45]. The reduction was larger in the first 5 years of follow up (35 vs 89 cancers, HR = 0·39, (0·27–0·58), *p* < 0·0001), but was still significant in the post 5-year period (50 vs 76 new cases, HR = 0·64, (0·45–0·91), *p* = 0·014), and was not significantly different from the first 5 years (*p* = 0·087). Invasive ER+ breast cancer was reduced by 54% (HR 0·46, (0·33–0·65), *p* < 0·0001). A 59% reduction in ductal carcinoma in situ was also observed (0·41, 0·22–0·79, *p* = 0·0081), especially in participants known to be ER+ (HR = 0·22, (0.07–0·65), *p* < 0·0062). No significant difference in deaths has been observed overall (69 vs 70, HR 0·96, 95% CI 0·69–1·34, *p* = 0·82), or for breast cancer (two anastrozole vs three placebo). A significant decrease in incidence of non-breast cancers was also observed for anastrozole (147 vs 200, HR = 0·72, (0·57–0·91), *p* = 0·0042), owing primarily to less non-melanoma skin cancer. No excess of fractures or cardiovascular disease was observed. Further follow-up is needed to assess the effect on breast cancer mortality.

For exemestane a total of 4560 postmenopausal women at increased risk of breast cancer, aged >35 years (median age 62.5 years) and a median Gail 5-year risk score of 2.3% were randomly assigned to either exemestane (2285) or placebo (2275) in the NCIC Clinical Trials Group Prevention.3 Trial (MAP.3 trial) [46]. After a median follow-up of 35 months, 11 invasive breast cancers were detected in the exemestane arm and 32 in those given placebo, resulting in a 65% relative reduction in invasive breast cancer (HR = 0.35, (0.18–0.70), *P* = 0.002), If DCIS is also included, there were 20 cancers in the exemestane arm and 44 for placebo (HR = 0.47 (0.27 to 0.79; *P* = 0.004). During this short follow-up period, exemestane was not associated with any serious toxic effects and only minimal changes in health-related quality of life. More work is needed to better determine exemestane’s longer term effectiveness and role in prevention.

The tolerability of aromatase inhibitors is substantially poorer and drop-out greater in the adjuvant trials of aromatase inhibitors than in the IBIS-II and MAP-3 prevention trials which begs the question of why? It is possible that the healthier status of women in a prevention trial might lead to their being more tolerant but given that most women with early breast cancer are otherwise well makes this an unlikely explanation. An alternative is that women with breast cancer may have a heightened state of anxiety and be more susceptible to tolerability issues because of that. An attractive hypothesis is that some women that develop ER+ breast cancer do so in part because of a more oestrogenic physiologic state and that this also renders them more sensitive to the side-effects of oestrogen deprivation. The population of such women in prevention trials would be “diluted” by those that do not share that phenotype. The presence of a greater oestrogenic state is supported by the observations on free-E2 discussed below and might also be enhanced by greater downstream sensitivity. We aim to test whether side-effects are greater in those women with higher baseline free-E2 levels in IBIS-II.

## Contemporary evidence on oestrogen-related risk of breast cancer

**Fig. 5:**
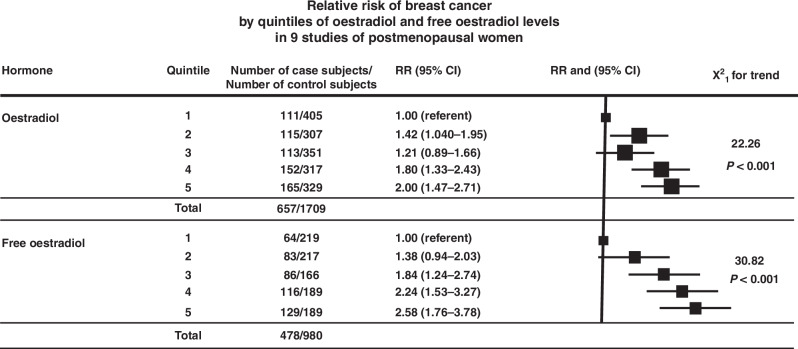
Key and colleagues in a combined analysis of 656 cases who developed cancer and 1709 women who didn’t in 9 studies [7], found that that the risk of breast cancer for women in the highest quintile of serum oestradiol was 2 fold higher than for those in the lowest quintile, with a clear dose response relationship (*p* < 0.00001, trend). For those who had free oestradiol measured the relative risk between lowest and highest quintiles was 2.58 (*p* < 0.00001, trend).

A comprehensive picture of the relationship between plasma oestradiol and breast cancer in postmenopausal women has been provided by Key [7] by producing a combined analysis of 9 studies. This analysis showed that the risk of breast cancer for women in the highest quintile of serum oestradiol was 2.00 fold higher compared those in the lowest quintile, with a clear dose response relationship (Fig. 5, p = 0.00004). There was an even stronger effect for free oestradiol, ie serum oestradiol that was not bound to SHBG or albumin (relative risk = 2.58, p < 0.00001, Fig. 5). This stronger effect is consistent with the understanding that it is only the protein-free fraction of oestradiol that can pass across the cell membrane and is therefore biologically active. However, free oestradiol is difficult to measure and the ratio of total oestradiol to SHBG in often used as a surrogate. A strong relationship was also found in this study between plasma testosterone levels and breast cancer risk, and this has been confirmed in later studies and is probably due to its being the immediate substrate for conversion to oestradiol by aromatase.

The relationship of plasma oestrogens and breast cancer in premenopausal women is much less clear, and any relationship is much weaker than for postmenopausal women, probably at least partly due to the large variations in endogenous oestrogen levels during the menstrual cycle [47–49].

## Interaction of plasma oestradiol and breast cancer prevention strategies

The association of plasma oestradiol levels with breast cancer risk prompts the question of whether preventive agents that target the pathway of oestrogenic tissue stimulation might be more effective in those women with the highest plasma oestradiol levels. This concept is supported by the observation that, in the Multiple outcomes for raloxifene evaluation (MORE) trial of the SERM raloxifene in women with osteoporosis, the decrease in relative risk of breast cancer was greatest in those in the highest tertile of plasma oestradiol [50]. However, in the NSABP-P1 study of tamoxifen in a high breast cancer risk population, against expectations, there was no association of oestradiol or SHBG with the risk of developing breast cancer and no indication of an interaction of these with tamoxifen’s preventive efficacy [51]. Our recent data with the aromatase inhibitor, anastrozole in the IBIS-II trial are much more supportive of women with a higher oestrogenic environment being more responsive to breast cancer prevention with an aromatase inhibitor [52]. We are currently assessing whether a similar relationship is evident with tamoxifen in the IBIS-I trial.

**Fig. 6:**
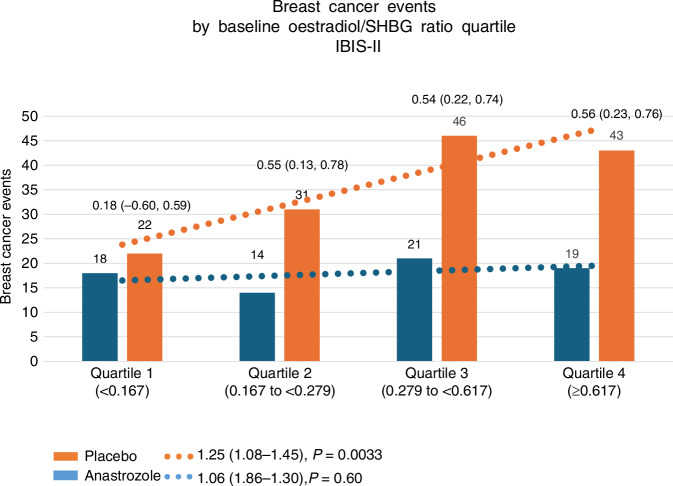
The case-control study included 212 participants from the anastrozole group (72 cases, 140 controls) and 416 from the placebo group (142 cases, 274 controls). A trend of increasing breast cancer risk with increasing E2/SHBG ratio was found in the placebo group (trend per quartile 1·25 (1·08–1·45), *p* = 0·0033), but not in the anastrozole group (HR = 1·06 (0·86–1·30), *p* = 0·60).

In IBIS-II, participants had blood taken at baseline, and 1 and 5-year follow-up visits. To date only the baseline samples have been analysed for women who developed cancer (N = 72 cases receiving anastrozole;144 cases receiving placebo) and compared with two matched controls per case [52]. The assays were done by Dr Brian Keevil in Manchester with oestradiol and testosterone being measured by Liquid chromatography tandem mass spectrometry (LC/MS-MS) [53]. In this pre-planned study, the primary endpoint was the effect of the anastrozole on all breast cancers, including ductal carcinoma in situ via a reduction of unbound oestradiol, as assessed by the oestradiol/SHBG (E2/SHBG) ratio. Cases were participants in whom breast cancer was reported after trial entry but before the follow up cutoff on Oct 22, 2019, and who had valid blood samples and no use of hormone replacement therapy within 3 months of trial entry or during the trial. For each case, two controls without breast cancer were selected at random, matched on treatment group, age (within 2 years), and follow-up time (at least that of the matching case). For each treatment group, a multinomial logistic regression likelihood-ratio trend test was applied to assess what change in the proportion of cases was prevented by a one-quartile change in the E2/SHBG ratio. Controls were used only to determine quartile cutoffs. A secondary analysis investigated the effect of the baseline testosterone/SHBG ratio on breast cancer development. After exclusions, the case-control study included 212 participants from the anastrozole group (72 cases, 140 controls) and 416 from the placebo group (142 cases, 274 controls). A trend of increasing breast cancer risk with increasing E2/SHBG ratio was found in the placebo group (trend per quartile 1·25 [1·08–1·45], *p* = 0·0033), but not in the anastrozole group (HR = 1·06 [0·86–1·30], *p* = 0·60; Fig. 6). Thus when compared to placebo a significant reduction of risk with anastrozole was seen in quartile 2 (HR = 0·55 (0·13 to 0·78]), quartile 3 (0·54 [0.22–0.74], and quartile 4 (0·56 [0·23 to 0.76]). These findings indicate that the benefit of anastrozole in reducing the risk of developing breast cancer during this follow-up period was approximately 50% for the 75% of women whose E2/SHBG was in the top three quartiles, At this stage it is unclear if risk for the highest quartile of E2/SHBG is higher than for the second or third quartile, or if the there is a risk threshold somewhere in or near the second quartile of E2/SHBG. More data is needed to determine this.

However, given that breast cancer is the commonest cancer in women, even these incomplete findings suggest that serum oestradiol should be measured more routinely and integrated into risk management decisions. A good time to make the first measurement would be alongside the first mammographic screening test taken after postmenopausal status is assured. Measuring serum concentrations of oestradiol and SHBG is inexpensive and can help clinicians decide which women are likely to benefit most from using an aromatase inhibitor in the preventive setting.

## Summary and conclusions

Over the last 4 decades a number of large clinical trials have convincingly demonstrated that tamoxifen reduces the risk of breast cancer in high-risk women in both premenopausal and postmenopausal populations. While tamoxifen remains the agent of choice in premenopausal women, aromatase inhibitors are more effective and have fewer serious side-effects in postmenopausal women. Improving the efficacy-to-tolerability ratio remains a key objective to increase the uptake of these drugs for preventive purposes. For tamoxifen this may be achievable by using lower doses than used for treating established breast cancer. For aromatase inhibition, the plasma E2/SHBG ratio should be included in risk profiling to identify a level with an enhanced therapeutic ratio. Oestradiol and SHBG concentrations can now be easily and accurately measured. More studies are needed to determine the optimal dose, duration of treatment and choice of AI to minimize both breast cancer risk and side effects on an individual level. IBIS-II was carried out on women who had other risk factors for breast cancer and it will be necessary to evaluate what approach is best for women without other known risk factors and how the E2/SHBG ratio can be integrated into the Tyrer-Cuzick model. The gold standard to establish this modified prevention paradigm would be a randomised clinical trial in which women are recruited with a high E2/SHBG ratio as one of their risk factors. A stepping stone might be provided by retrospectively determining baseline E2/SHBG levels in women that do or do not develop contralateral breast in adjuvant trials of aromatase inhibitors if the necessary samples exist. The novel approach of Decensi in conducting presurgical studies to assess potential approaches to breast cancer prevention could also contribute to taking this new approach to clinical practise.

Measuring oestradiol level and controlling it with an aromatase inhibitor may be the first opportunity to manage a potential cancer in the way cardiologists deal with heart disease by measuring cholesterol and using a statin to lower it when necessary. In addition to trials to determine the best choice of treatment, work will also be necessary to find the best way of integrating them into the health care system.

This approach of concentrating on lowering free oestradiol levels in higher risk postmenopausal women could have a large impact on the occurrence of the commonest cancer in women and needs to be fully investigated.
